# Supplementary material for: Extratubular Polymerized Uromodulin Induces Leukocyte Recruitment and Inflammation In Vivo
Source: Front Immunol. 2020 Dec 22;11:588245. doi: 10.3389/fimmu.2020.588245 (PMC7783395; doi:10.3389/fimmu.2020.588245)
Supplement: Supplementary file 1 [file DataSheet_1.pdf]

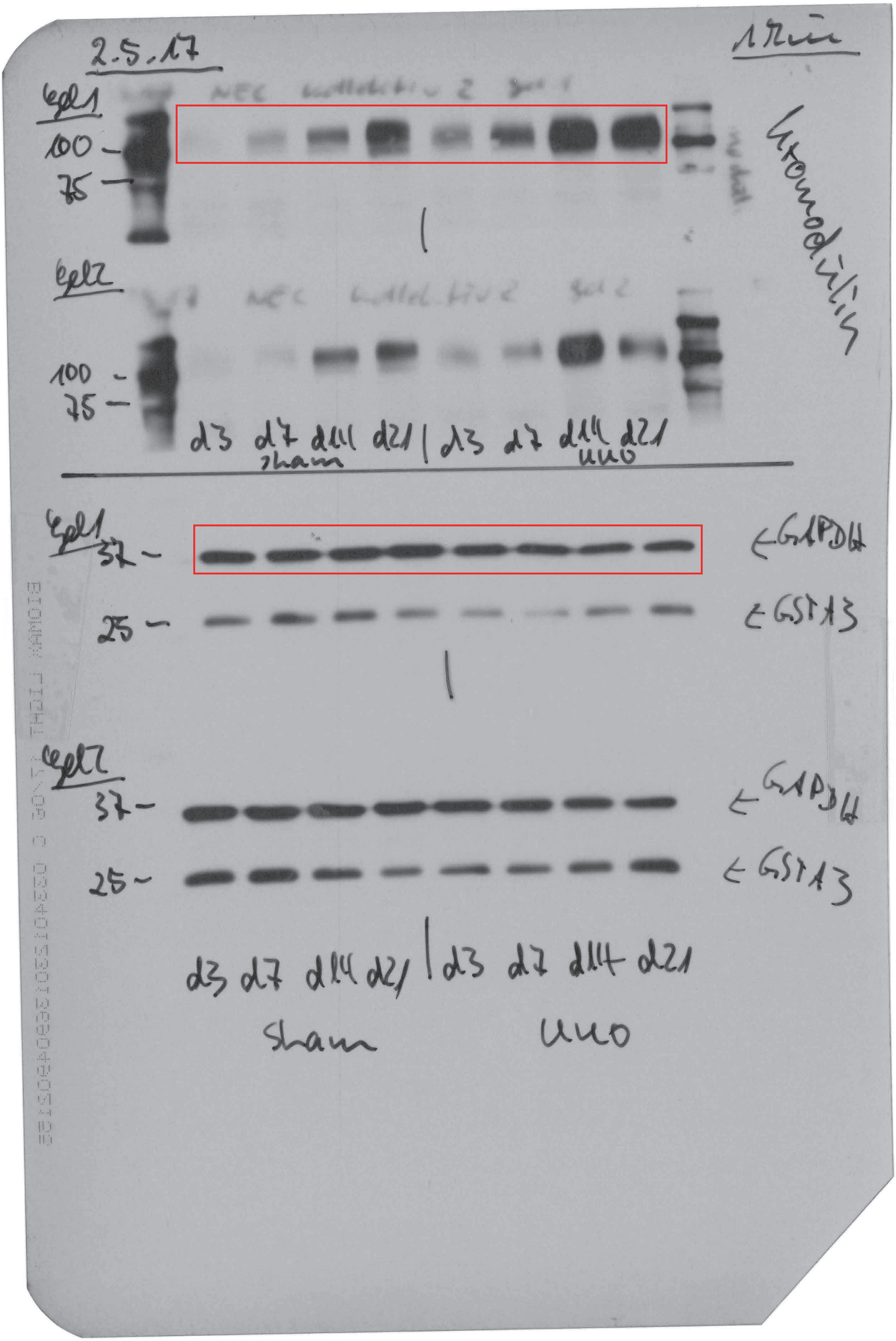
2.5.17
1min
Gel1
100
75
Uromodulin
Gel2
100
75
d3 d7 d14 d21 | d3 d7 d14 d21
sham
UUO
Gel1
37
25
← GAPDH
← GSTA3
Gel2
37
25
← GAPDH
← GSTA3
d3 d7 d14 d21 | d3 d7 d14 d21
sham
UUO
