# Supplementary figures and images for: Extratubular Polymerized Uromodulin Induces Leukocyte Recruitment and Inflammation In Vivo
Source: Front Immunol. 2020 Dec 22;11:588245. doi: 10.3389/fimmu.2020.588245 (PMC7783395; doi:10.3389/fimmu.2020.588245)

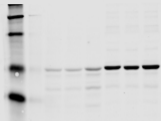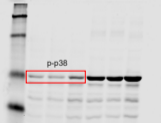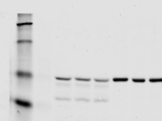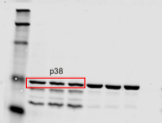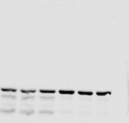

Supplement: Supplementary file 3 [file DataSheet_3.pdf]
